# The National Institutes of Health COVID-19 NeuroDatabank and NeuroBiobank: A National Resource for Learning, Discovery, and Progress

**DOI:** 10.3389/fneur.2020.615061

**Published:** 2021-01-15

**Authors:** Andrea B. Troxel, Jennifer A. Frontera, Carolina Mendoza-Puccini

**Affiliations:** ^1^Department of Population Health, New York University Grossman School of Medicine, New York, NY, United States; ^2^Department of Neurology, New York University Grossman School of Medicine, New York, NY, United States; ^3^Division of Clinical Research, National Institute of Neurological Disorders and Stroke, Bethesda, MD, United States

**Keywords:** COVID-19, registry, neurology, epidemiology, patient privacy, rare conditions

## Abstract

Patients suffering from COVID-19 experience a wide range of symptoms and sequelae, including increasingly recognized neurological problems. A concerted effort is necessary to identify and characterize these issues, whether newly appearing as a result of COVID-19 disease or exacerbations of underlying conditions. A national resource to collect information and/or biospecimens regarding neurological complications of COVID-19 offers an opportunity for broad representation, harmonization, and rapid learning, all while ensuring robust protection of confidential information through the use of global unique identifiers to protect patient privacy.

## Introduction

There is accumulating evidence of neurological complications of COVID-19, but their prevalence, etiology, and long-term cognitive and functional sequelae remain unknown. The current neurologic COVID-19 literature consists primarily of retrospective studies that often conflate non-specific symptoms (e.g., agitation, executive dysfunction, myalgias, dizziness, and headache) with neurologic diagnoses (e.g., stroke, seizures, and Guillain-Barre syndrome), leading to wide variability in neurologic event prevalence estimates (4–84% across studies) (1–3). While initial reports speculated on the neuro-invasiveness of SARS-CoV-2, no convincing pathologic data exist to support neurotropism (4). Conversely, case reports of Guillain-Barre syndrome and acute disseminated encephalitis suggested post-infectious, autoimmune-mediated neurological injury. Furthermore, an emerging literature describing a post-viral syndrome characterized by fatigue, cognitive problems, and neuropsychiatric disorders points to a potential second wave of subacute COVID-19-related neurological conditions (5). It remains unclear whether neurologic disorders in the context of COVID-19 represent a causal relationship, secondary effects of severe systemic illness, or mere coincidence.

Local medical and research institutions across the world have generated datasets with information on COVID-19 patients and have collected biosamples of, for example, blood, plasma, cerebrospinal fluid, and placenta and brain tissue. However, most such resources were established hurriedly with little to no funding or staff and often missed the peak of the pandemic in their regions. Furthermore, data on symptoms, tests, treatments, and outcomes of patients with COVID-19 exist in the idiosyncratic electronic health records (EHRs) of individual hospitals and clinics. Collectively, this information has the potential to accelerate research, enable learning about the prevalence and consequences of COVID-19 complications, and facilitate the development of prevention and treatment strategies. But its current fragmentary state hampers scientific progress. An urgent challenge is to assemble, harmonize, and curate these diverse resources and make them widely available to researchers.

The practical challenges of establishing harmonized data collection and biobanking are formidable, and include ownership as well as data security, privacy, harmonization, and standards. Here we discuss ways to overcome these challenges in the context of neurological complications of COVID-19, although our experience can be applied in other domains.

The NIH COVID-19 NeuroDatabank and NeuroBiobank (NIH-NeuroCOVID), funded in July 2020 by the National Institute of Neurological Diseases and Stroke (NINDS), was developed as a resource for investigators interested in pooled COVID-19 neurological event data. The main goals of the program are to identify neurologic phenotypes, risk factors, regional effects, socio-economic factors, and therapeutic responses among patients with new or complicated neurological disorders and concomitant COVID-19. Numerous features of the NIH-NeuroCOVID initiative enhance its promise as a basis for rapid accumulation of knowledge, sharing of harmonized data and curated information, and leveraging of geographically and socially diverse populations to accelerate real-time learning.

## Methodology

### Inclusion/Exclusion Criteria

The databank will include both hospitalized patients and outpatients across the age spectrum including maternal/neonate (birth to 30 days of life) dyads, children, pregnant women, and the general adult population. Inclusion criteria are laboratory confirmed SARS-CoV-2 infection (either by RT-PCR molecular testing, antigen, or antibody testing) and at least one new or worsened symptom related to the nervous system.

### Data Curation

A key feature supporting the power of the program as an engine of inquiry is the standardization of data elements that may arise from a variety of sources into a set of defined common data elements (CDEs). The NeuroCOVID data management system will include robust quality control measures, with a limited set of critical required elements and a much wider set of optional elements; this will ensure that the most important information is captured uniformly while enabling ancillary studies of a broader range of topics.

Data collection will follow a standardized format and systematic coding, to maximize our ability to represent information in a consistent manner. Building upon standardized NIH/NINDS common data elements (CDEs), we created additional variables to capture COVID-19-specific medical complications as well as new or worsened neurological disorders. The harmonization of variable definitions, capturing aspects of infection, disease course, treatment, outcomes, and complications, will enable analyses that define the scope of the problem, indicate associations that may be important for treatment and management, and present further avenues for examination. To assess causality, we adapted previously published association criteria developed for COVID-19 related meningitis, encephalitis, central nervous system vasculitis, and myelitis (3). We included additional variables to establish the timing from COVID-19 diagnosis or initial symptom onset. For ease of use and generalizability, we will use the World Health Organization Clinical (WHO) Progression Score to determine COVID-19-related severity of illness and the modified Rankin Score (in adults) and the Pediatric Functional Status Scale (in neonates and children) to assess neurological function at the time of hospital discharge or outpatient presentation. Other hospital metrics include the NIH stroke score, Glasgow Coma score, sequential organ failure assessment (SOFA), Pediatric Logistic Organ Dysfunction Score (PELOD-2), Confusion Assessment Method (CAM or CAM-ICU), length of stay, intensive care unit requirement, ventilator days, and discharge disposition. All of these instruments are among the most commonly documented scoring systems utilized by health systems across the U.S.

### Data Security

To enable widespread sharing of the resources created by NeuroCOVID, we have established robust processes for de-identification of both data and biospecimens, with protection of patient privacy as a guiding principle. All data and samples will be stripped of personal identifiers and linked only to a Global Unique Identifier (GUID); this process enforces retention of personal protected health information only at the originating site (one-way encryption), and enables linking of patient data from multiple sources, and between pregnant mothers and their newborn infants. Dates and other potentially identifying elements are converted into a series of hash-codes using a specialized algorithm to ensure non-identifiability and the inability to link back to individuals following submission of data. This robust process of de-identification will ensure the creation of fully anonymized datasets that can then be widely, and securely, shared with researchers, in full accordance with NIH data sharing policies and goals.

### Governance

We have created a web-based portal to accept applications to use NeuroCOVID resources (https://med.nyu.edu/departments-institutes/population-health/divisions-sections-centers/biostatistics/research/nih-neurodatabank-neurobiobank). Research proposals to use data and/or biospecimens will be uploaded via a streamlined interface. Our Steering Committee, comprising international experts in neurology, infectious disease, and biostatistics and epidemiology, will assess these proposals with respect to scientific rigor, adequate design and statistical power, suitability of analytic methods, and appropriate plans for dissemination. Proposals of sufficient quality and scientific merit will be approved and investigators will be provided with the requested materials. We have developed publication policies to appropriately credit contributors. We will provide researchers who use NeuroCOVID data with support to encourage application of appropriately robust methods for the analysis of observational data (e.g., estimation of propensity scores followed by matching to render groups more comparable) to address the problems with selection bias and other forms of confounding that are inherent in observational data sources (6).

## Discussion

The NIH COVID-19 NeuroDatabank will harness the power of pooling information, so critical in a rapidly evolving pandemic (7). This has multiple advantages. Foremost is the ability to incorporate data from a truly diverse population of patients, including underrepresented, vulnerable populations, whether they be defined by race/ethnicity, socioeconomic status, or social and/or physical marginalization (e.g., rural, homeless, or justice-system-involved populations). While we cannot guarantee the representativeness of the data collected, a strong effort will be made to enroll a diverse patient population and encourage participation by a variety of institutions and practitioners. A second advantage is the ability, by casting a wide net across the nation and potentially the globe, to uncover and document rare effects that are impossible to detect with less far-ranging or more focused sampling. This effort in data collection will enable a rich characterization of the emerging neurological effects of COVID-19, and just as importantly, of the impact of the infection on existing or latent neurologic illnesses.

An additional unique feature of NIH-NeuroCOVID is the pairing of the comprehensive national NeuroDatabank with the NeuroBiobank, a resource that will accept, catalog, store, and track biospecimens from COVID-19 patients with neurological symptoms. The samples stored and tracked will constitute a rich resource, available to researchers following a simple application process, for the study of mechanistic questions, epidemiological associations, and potential therapies. The ability to link the granular demographic, clinical, and social data collected in the NeuroDatabank with the biospecimen material housed in the NeuroBiobank provides an important opportunity for learning and discovery.

NIH-NeuroCOVID will not replace efforts to identify effective treatments via well-designed and conducted RCTs. There is no substitute for gold-standard RCT evidence (8). But NIH-NeuroCOVID offers an important adjunct, which may be more agile and able to quickly provide critical pieces of information.

## Conclusion

The NIH COVID-19 NeuroDatabank and NeuroBiobank constitute an important national resource with robust infrastructure, quality control measures, and assurance of patient privacy and confidentiality. They address the mandate for comprehensive inclusion of diverse populations; indeed, widespread participation and uptake are crucial to their success. The resources provide a critical foundation for the generation of hypotheses and ideas for management and treatment of COVID-19 and its neurological complications, and the evaluation of those hypotheses with harmonized and representative data. The initiative offers a model for responding to public health crises that will undoubtedly arise in the future to test our national capacity and capability as stewards of public health and wellness.

## Data Availability Statement

The original contributions presented in the study are included in the article/supplementary material, further inquiries can be directed to the corresponding author/s.

## Author Contributions

AT, JF, and CM-P contributed equally to conception and writing of the manuscript. All authors contributed to the article and approved the submitted version.

## Conflict of Interest

The authors declare that the research was conducted in the absence of any commercial or financial relationships that could be construed as a potential conflict of interest.
